# Oxidative cyclization of alkenols with Oxone using a miniflow reactor

**DOI:** 10.3762/bjoc.5.18

**Published:** 2009-04-29

**Authors:** Yoichi M A Yamada, Kaoru Torii, Yasuhiro Uozumi

**Affiliations:** 1 RIKEN, Hirosawa, Wako, Saitama 351-0198, Japan; 2 Institute for Molecular Science (IMS), Myodaiji, Okazaki, Aichi 444-8787, Japan

**Keywords:** alkenols, cyclic ethers, miniflow reaction, oxidative cyclization, Oxone

## Abstract

A miniflow system for oxidative cyclization of alkenols with Oxone was developed. Thus, the oxidative cyclization of (*Z*)- and (*E*)-alkenols in *i*-PrOH with an aqueous solution of Oxone proceeded smoothly and safely in a PTFE tube without any exogenous catalytic species, and was subsequently quenched in a flow-reaction manner to afford the corresponding furanyl and pyranyl carbinols quantitatively within 5 or 10 min of residence time.

## Introduction

The development of flow-reaction systems for molecular transformations is an important goal in organic syntheses. Recently, innovative devices such as micro- and miniflow reactors that offer many fundamental as well as practical advantages for efficient organic transformations have been gaining ground in chemical experimentation [1–15]. Extensive investigations have revealed that the large interfacial area and the short molecular diffusion path in narrow space reactors often drastically improve the efficiency of a given chemical reaction. As a case in point, we have previously developed a catalyst-installed microflow reactor where a membranous polymeric palladium catalyst was deposited inside a micro-channel reactor at the laminar flow interface [16], resulting in the instantaneous production of biaryls (quantitative yield within 4 s of residence time) via a palladium-catalyzed Suzuki-Miyaura reaction under microflow conditions. An additional advantage of micro- and minireactors is the small heat capacity of the micro- and miniflow systems thus rendering exothermic and/or potentially explosive reactions safe and practical. Consequently, oxidative transformations with potentially explosive oxidants would be ideal target reactions for miniflow systems. We wish to report the oxidative construction of furanyl and pyranyl alkyl carbinols with Oxone via a miniflow reaction system.

## Results and Discussion

Furanyl and pyranyl carbinols have generated considerable interest due to their presence in a number of therapeutically and biologically active compounds [17–31]. We therefore decided to turn our attention to developing an oxidative cyclization of alkenols [32–33] for the preparation of furanyl and pyranyl alkyl carbinols. During our investigation, we found that the oxidative cyclization of (*Z*)-4-decen-1-ol (**1a**) with Oxone (2KHSO_5_·KHSO_4_·K_2_SO_4_) proceeded at 80 °C without any exogenous catalysts under small-scale batch conditions (up to 50 mmol of **1a**) to give *threo*-1-(2-tetrahydrofuranyl)hexan-1-ol (**2a**) in 99% yield within 5 min (Table 1) [34]. When a mixture of an aqueous solution of Oxone (100 mM, 1 mL, 2 equiv vs **1a**) and a 2-propanol solution of **1a** (50 mM, 1 mL) was stirred at 80 °C for 5 min, the cyclization took place very smoothly to afford *threo*-1-(2-tetrahydrofuranyl)hexan-1-ol (**2a**) in 99% yield as a single racemic diastereoisomer. Yet when 30% aq H_2_O_2_ was used as the oxidant at 80 °C, the cyclization hardly proceeded at all, even with a longer reaction time [32]. We had previously found that a polymeric phosphotungstate catalyst promoted the cyclization of **1a** with 30% aq H_2_O_2_ at 50 °C with a much longer reaction time (24 h). Thus, Oxone was found to be the most efficient oxidant to promote the cyclization of (*Z*)-4-decen-1-ol (**1a**). Although a powerful and inexpensive oxidant for this transformation [35–42], Oxone however is also a known fire and explosion hazard [43] essentially rendering its large-scale use impractical. To avoid these potentially dangerous and hazardous conditions in a large-scale batch oxidation, we switched the conventional batch system to a miniflow system.

**Table 1:** The oxidative cyclization of an alkenol **1a** with Oxone under batch conditions.

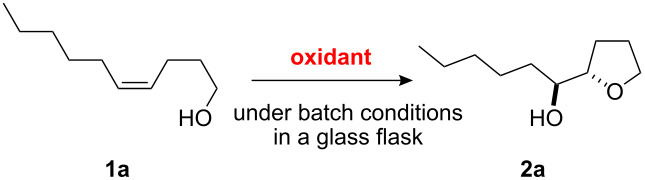		
oxidant	conditions	yield of **2a** (%)

aq Oxone	80 °C, 5 min	99
30% aq H_2_O_2_	80 °C, 60 min	no reaction
polymeric PW_12_O_40_^3−^ (cat) with 30% aq H_2_O_2_ (see [32])	50 °C, 24 h	99

**Figure 1:**
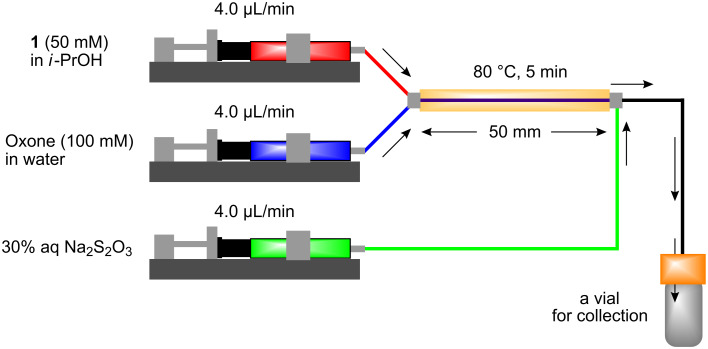
Miniflow reaction system of oxidative cyclization.

The miniflow reaction system is composed of poly(tetrafluoroethylene) (PTFE) tubes of ø = 1 mm, T-shaped connectors, and syringes with syringe pumps as shown in Figure 1. When the miniflow reaction of the alkenols **1** in *i*-PrOH with an aqueous solution of Oxone was carried out in the miniflow reactor with 5 min of residence time at 80 °C, we were pleased to see that the reaction proceeded smoothly to afford the corresponding cyclic ethers **2** in high conversion. Thus, a solution of an alkenol **1** in *i*-PrOH (50 mM) and Oxone in water (100 mM) were oppositely injected with a flow rate of 4.0 μl/min each by using syringe pumps from the individual inlets. The mixed solution passed through a PTFE tube reactor (length = 50 mm) at 80 °C, and then was quenched with 30% aq Na_2_S_2_O_3_ solution injected into the flow tube with a flow rate of 4.0 μl/min. The resulting organic/aqueous outflow was collected in a glass vial. The chemical conversion and structure of the products were determined by GC and ^1^H NMR analysis. As shown in Table 2, entry 1, the oxidative cyclization of (*Z*)-4-decen-1-ol (**1a**) with Oxone was performed within 5 min of residence time to afford *threo*-1-(2-tetrahydrofuranyl)hexan-1-ol (**2a**) in 99% conversion. The cyclization of (*Z*)-4-hexen-1-ol (**1b**) and (*Z*)-4-hepten-1-ol (**1c**) proceeded smoothly to give the *threo*-tetrahydrofuranyl alcohols **2b** and **2c** in 90% and 88% conversion, respectively (entries 2 and 3). This flow reaction system was also utilized for the formation of six-membered cyclic ethers. Thus, the oxidation of (*Z*)-5-octen-1-ol (**1d**) was carried out with a flow rate of 2.0 μL/min (residence time: 10 min) to give 90% conversion of the *threo*-tetrahydropyranyl alcohol **2d** (entry 4). (*E*)-4-Decen-1-ol (**1e**) underwent oxidative cyclization with 10 min residence time to afford the *erythro*-product **2e** in 70% conversion (entry 5). These stereochemical observations indicate that the cyclization involves a stereospecific reaction pathway. The reaction pathway of the present oxidative cyclization should proceed via the epoxidation of the alkene **1** with Oxone and subsequent oxirane ring opening with the intramolecular oxygen nucleophile (an intramolecular S_N_2 reaction) to afford the product **2** stereospecifically [44–45]. It should be noted that the miniflow cyclization of **1a** was continuously carried out to give a quantitative conversion of **2a** over 2 h.

**Table 2:** Oxidative cyclization of alkenols with Oxone through a miniflow reactor.^a^

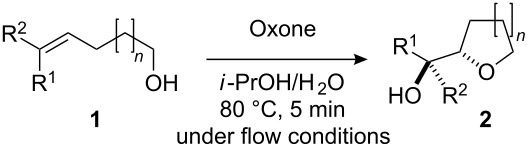			
Entry	Substrate	Product	Conversion (%)

1	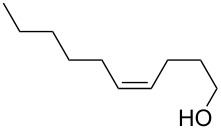 **1a**	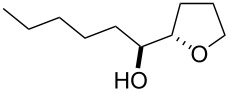 **2a**	99
2	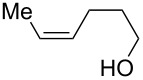 **1b**	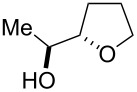 **2b**	90
3	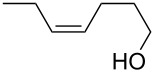 **1c**	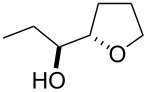 **2c**	88
4^b^	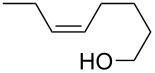 **1d**	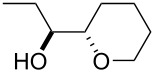 **2d**	90
5^b^	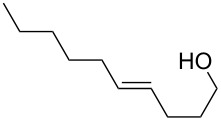 **1e**	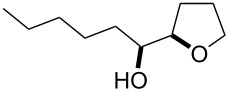 **2e**	70

^a^alkenol (50 mM in *i*-PrOH), Oxone (2KHSO_5_·KHSO_4_·K_2_SO_4_) (100 mM in H_2_O), flow rate: 4.0 μL/min each, 80 °C, residence time = 5 min; several fractions were collected for each reaction shown to demonstrate the stable and high reactive performance of the miniflow reactor; the product **2a** was obtained in 0.12 mmol/h. ^b^flow rate: 2.0 μL/min each, 80 °C, residence time = 10 min.

## Conclusion

In conclusion, we have developed a miniflow reaction system for the oxidative cyclization of alkenols with Oxone, affording the corresponding cyclic ethers in high conversion, where potentially explosive Oxone was used and quenched safely. Development of instantaneous flow reaction systems for the oxidation reactions with a retention time of several seconds is currently in progress.
